# ATP is an essential autocrine factor for pancreatic β‐cell signaling and insulin secretion

**DOI:** 10.14814/phy2.15159

**Published:** 2022-01-10

**Authors:** Sebastian Hauke, Jona Rada, Gergely Tihanyi, Danny Schilling, Carsten Schultz

**Affiliations:** ^1^ Cell Biology & Biophysics Unit European Molecular Biology Laboratory (EMBL) Heidelberg Germany; ^2^ Department of Chemical Physiology and Biochemistry Oregon Health & Science University (OHSU) Portland Oregon USA

**Keywords:** apyrase, Ca^2+^ signaling, extracellular, oscillations

## Abstract

ATP has been previously identified as an autocrine signaling factor that is co‐released with insulin to modulate and propagate β‐cell activity within islets of Langerhans. Here, we show that β‐cell activity and insulin secretion essentially rely on the presence of extracellular ATP. For this, we monitored changes of the intracellular Ca^2+^ concentration ([Ca^2+^]_i_ oscillations) as an immediate read‐out for insulin secretion in live cell experiments. Extensive washing of cells or depletion of extracellular ATP levels by recombinant apyrase reduced [Ca^2+^]_i_ oscillations and insulin secretion in pancreatic cell lines and primary β‐cells. Following ATP depletion, [Ca^2+^]_i_ oscillations were stimulated by the replenishment of ATP in a concentration‐dependent manner. Inhibition of endogenous ecto‐ATP nucleotidases increased extracellular ATP levels, along with [Ca^2+^]_i_ oscillations and insulin secretion, indicating that there is a constant supply of ATP to the extracellular space. Our combined results demonstrate that extracellular ATP is essential for β‐cell activity. The presented work suggests extracellular ATPases as potential drug targets for the modulation of insulin release. We further found that exogenous fatty acids compensated for depleted extracellular ATP levels by the recovery of [Ca^2+^]_i_ oscillations, indicating that autocrine factors mutually compensate for the loss of others. Thereby, our results contribute to a more detailed and complete understanding of the general role of autocrine signaling factors as a fundamental regulatory mechanism of β‐cell activity and insulin secretion.

## Graphical abstract

We find that ATP is an essential extracellular signaling component for calcium oscillations and insulin secretion from beta‐cells. Removal of ATP or blocking of P2Y receptors will stop oscillations. This can be partially compensated by adding fatty acids that stimulate calcium signaling via GPR40.

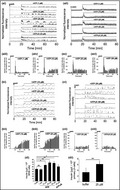

## 1 INTRODUCTION

Pancreatic β‐cells are biological oscillators that respond to extracellular glucose with continuous oscillations of the intracellular Ca^2+^ concentration ([Ca^2+^]_i_ oscillations). Integration of the pancreatic β‐cell population into functional syncytia provides synchronization of [Ca^2+^]_i_ oscillations along with periodic secretory bursts of insulin from the islets of Langerhans (Bergsten et al., 1994; Goodner et al., 1977; Lefebvre et al., 1987; Pørksen et al., 1995, 2002; Santos et al., 1991; Shapiro et al., 1988). The basic mechanism for β‐cell synchronization relies on direct gap‐junctional coupling of β‐cells by connexins (Calabrese et al., 2003; Meda et al., 1979; Meissner, 1976; Orci et al., 1973). However, loss of functional gap junctions or dissociation of pancreatic islets into single β‐cells was shown to compromise, but not to abolish synchronized [Ca^2+^]_i_ oscillations and pulsatile insulin release (Ravier et al., 2005; Squires et al., 2000). Therefore, intercellular synchronization was suggested to be also mediated by small diffusible ligands of G‐protein‐coupled receptors (GPCRs) in the extracellular space of pancreatic islets, in addition to gap junctions (Grapengiesser et al., 1999; Squires et al., 2002). Para‐ and autocrine signaling is based on the microcirculation of small diffusive extracellular messengers, mediating effects on either cells from which they have been released as well as on neighboring cells of the same type. This allows for information exchange about the functional state of neighboring cells and for the synchronization of β‐cells within pancreatic islets (Braun et al., 2012; Caicedo, 2013; Tengholm & Gylfe, 2009). The cellular arrangement and the confined extracellular space along with short distances between communicating cells inside islets of Langerhans are perfectly suited for efficient auto‐ and paracrine signaling (Caicedo, 2013). Small molecule and ion insulin secretagogues such as NO (Grapengiesser et al., 2001; Hellman et al., 2000), CO (Lundquist et al., 2003), Zn^2+^ (Richards‐Williams et al., 2008), neuropeptide Y (Wang et al., 1994), glutamate (Cabrera et al., 2008), γ‐aminobutyric acid (GABA) (Franklin & Wollheim, 2004), and ATP (Grapengiesser et al., 2004; Hellman et al., 2004) have been described as autocrine signaling factors for intra‐ or inter‐islet communication and β‐cell synchronization.

In our previous work, we demonstrated that lowering levels of autocrine signaling factors, in particular fatty acids (FAs) in β‐cell medium by stringent washing or by the application of fatty acid‐free bovine serum albumin (FAF‐BSA) immediately reduced glucose‐stimulated [Ca^2+^]_i_ oscillations and insulin secretion (Hauke et al., 2018). We found that endogenous FAs not only stimulate β‐cells, but are also secreted by these cells (Hauke et al., 2018). Following washing, transfer of supernatant from another population of MIN6 cells immediately fully reinstalled [Ca^2+^]_i_ oscillations (Hauke et al., 2018). Thereby, we not only demonstrated the essential role of extracellular autocrine signaling factors for β‐cell activity and insulin secretion, we also exploited the well documented correlation between [Ca^2+^]_i_ oscillations and insulin secretion (Bergsten, 1995; Gilon et al., 1993; Jonas et al., 1998).

Based on these observations, we suspected other cellular metabolites to function as essential autocrine signaling factors for β‐cell activity and insulin secretion. ATP is well known to be co‐released together with insulin from secretory granules that contain nucleotides in millimolar concentrations (Hutton et al., 1983; MacDonald et al., 2006; Wayne Leitner et al., 1975) resulting in local extracellular ATP concentrations of up to 25 µM at the cell surface (Hazama et al., 1998). Even higher extracellular concentrations of ATP might be reached by comparably small numbers of molecules that are released into the confined extracellular space of pancreatic islets (Braun et al., 2012; Caicedo, 2013). Acting on G‐protein‐coupled P2Y receptors and ionotropic P2X receptors, ATP increases [Ca^2+^]_i_ by stimulating InsP_3_‐mediated Ca^2+^ release from the endoplasmic reticulum (ER) (Khan et al., 2014) or by increased Ca^2+^ influx into the cell. External ATP was suggested to promote intra‐ and inter‐islet propagation of [Ca^2+^]_i_ oscillations in a para‐, and autocrine fashion within the enclosed pancreatic islet (Burnstock & Novak, 2013; Petit et al., 1998; Tengholm, 2014). Thereby, ATP entrains pancreatic islets into a common rhythm, to make islets sensitive to fluctuating glucose levels and to ensure rapid and adapted glucose responsiveness (Grapengiesser et al., 2004, 2005; Hellman et al., 2004; Jacques‐Silva et al., 2010; Petit et al., 2009). Extracellular ATP has been described to be rapidly cleared by plasma membrane‐bound ectonucleotidases with their active site facing to the extracellular milieu (Lavoie et al., 2010). Despite of data demonstrating the overall importance of autocrine ATP in the coordination and propagation of β‐cell activity across pancreatic lobes (Grapengiesser et al., 2004; Gylfe & Tengholm, 2014; Hellman et al., 2004), the role of extracellular ATP for individual β‐cell activity is yet to be determined.

In this study, we therefore aimed at (1) investigating the general role of ATP as an autocrine signaling factor for β‐cell activity and insulin secretion and (2) examining whether β‐cell activity can be modulated by the selective reduction or replenishment of extracellular ATP levels. Further, we (3) correlated increased levels of endogenous ATP in the presence of selective inhibitors of ecto‐ATPases with [Ca^2+^]_i_ oscillations and insulin secretion and (4) provide a possible mechanism for ATP‐mediated action.

## 2 RESEARCH DESIGN AND METHODS

### 2.1 Reagents

Dulbecco´s modified eagle medium (DMEM) were obtained from Gibco. β‐Mercaptoethanol (50 mM in PBS) was ordered from PAN Biotech (Aidenbach). Recombinant apyrase, ATP, stable ATP analogues, and Histopague 1083 (density: 1.083 g/mL) and Histopague 1119 (density: 1.119 g/mL) were purchased from Sigma‐Aldrich. Collagenase (from *Clostridium histolyticum*) was obtained from Nordmark Biochemicals. All other commercially available chemicals and enzymes were ordered from Sigma‐Aldrich.

### 2.2 Unit definitions of applied recombinant enzymes (according to the supplier, Sigma‐Aldrich)

*Apyrase* (from potato, enzyme commission [EC] number: 3.6.1.5, expressed in *Pichia pastoris*): One unit liberates 1.0 μmol of inorganic phosphate from ATP or ADP per minute at pH 6.5 and 30°C.

### 2.3 Culturing MIN6 cells

MIN6 cells (Miyazaki et al., 1990) were cultured in a humidified atmosphere at 37°C and 8% CO_2_. The culture medium DMEM contained 4.5 g/L glucose and was supplemented with FBS (15%, gradient‐grade, Thermo Fisher Scientific, catalog# 16000‐044) and β‐mercaptoethanol (70 µM). The medium was sterile‐filtered (Millex GV, 0.22 µm) and used within 1 week after preparation. For imaging, MIN6 cells were seeded into 8‐well LabTek microscope dishes (155411 Thermo Scientific) or on 40 mm coverslips (Menzel Gläser). For in vitro assays, MIN6 cells were seeded on ∅ 60 mm or ∅ 35 mm dishes (Nunc delta surface, cat# 150288/cat# 153066; Roskilde, Denmark) to form pseudoislets within 5 days after seeding. MIN6 cells were used exclusively from passages 26 to 36.

### 2.4 Culturing 1.1B4 cells

1.1B4 cells were cultured in a humidified atmosphere at 37°C and 8% CO_2_. The culture medium RPMI‐1640 medium (Merck) contained 2 g/L d‐glucose, 0.3 g/L l‐glutamine, and was supplemented with 10% FBS (gradient‐grade, Thermo Fisher Scientific, catalog# 16000‐044). The medium was sterile‐filtered (Millex GV, 0.22 µm) and used within 1 week after preparation. Cells were passaged at 80% confluency. For live cell [Ca^2+^]_i_ imaging, 1.1B4 cells were seeded into 8‐well LabTek microscope dishes (155411, Thermo Scientific). 1.1B4 cells were transfected with a plasmid encoding the Ca^2+^‐sensitive red‐fluorescent protein R‐Geco1 using lipofectamine 2000 (11668027, Thermo Scientific) 1 day prior to imaging experiments. The transfection was performed using cells attached to LabTek microscopy dishes at 60–80% confluency. After removing the medium and washing cells with DPBS, 210 μl OptiMEM medium was added to each well. Subsequently, 1.5 μl/well of lipofectamine 2000 (1 mg/ml) and 400 ng/well plasmid DNA were added to 20 μl/well OptiMEM medium, respectively. Both solutions were combined and incubated for 10 min at room temperature. 40 μl of this transfection mix were added to each well. After 6 h of incubation at 37°C in 8% CO_2_, OptiMEM was exchanged by 400 μl of RPMI‐1640 medium. Cells, showing a medium expression level of R‐Geco1, were used for live cell imaging the next day.

### 2.5 Isolation of mouse primary β‐cells

Female Ctrl: CD1 (ICR, outbred) mice (supplied by Charles River Laboratories, cat# CD1S1FE07W) served as donors for primary β‐cells that were isolated as previously described (Ravier & Rutter, 2010). In short, mice were sacrificed by cervical dislocation. A collagenase solution (1 mg/ml) was injected into the pancreatic duct, followed by extraction of the pancreas from the animal and digestion at 37°C for 10 min. A Histopaque gradient (1.083 and 1.119 g/ml) allowed for the isolation of pancreatic islets via density gradient centrifugation. Islets were incubated in RPMI medium (supplemented with 10% FCS, 100 U/ml penicillin, and 100 mg/ml streptomycin) for 24 h. Trypsin digestion (5 min, 37 °C) dissociated islets into single β‐cells that were seeded into LabTeks, pre‐coated with poly‐l‐lysine. Animals were housed in the EMBL animal facilities under veterinarian supervision and the guidelines of the European Commission, revised directive 2010/63/EU and AVMA guidelines 2007.

### 2.6 Mouse insulin ELISA

The quantification of insulin secretion was based on an enzyme‐linked immunosorbent assay (ELISA) in 96‐well format (Mercodia). Experiments for the determination of insulin secretion were performed in quadruplicate per condition, following the instructions of the supplier. Insulin levels were normalized to the protein content of MIN6 cells, as determined by a BCA assay (Pierce^TM^ BCA Protein assay kit, Thermo Scientific).

### 2.7 ATP bioluminescence assay

The quantification of extracellular ATP was based on a bioluminescence assay in 96‐well format. For the quantification of ATP from MIN6 supernatant, a firefly luciferase‐based assay was applied (serial number: A22066, Invitrogen). Experiments for the determination of extracellular ATP were performed in quadruplicate per condition, following the instructions of the supplier. ATP levels were normalized to cell numbers. For normalization of ATP levels in cellular supernatants, pre‐defined numbers of MIN6 or mouse primary β‐cells were correlated to cellular protein content following cell lysis, as determined by BCA assay. Then, cell numbers were inferred from determined protein content of harvested cells.

### 2.8 Confocal laser scanning microscopy

Live cell imaging was performed on a FluoView1200 (Olympus IX83) confocal laser scanning microscope, equipped with an environment box (made by EMBL) to allow imaging at 37°C and 5% CO_2_. Olympus 60x Plan‐APON (NA 1.4, oil) or 20x UPLS APO (NA 0.75, air) objectives and FluoView software (version 4.2) were applied. Images were acquired using a Hamamatsu C9100‐50 EM CCD camera. A 488‐nm laser line (120 mW/cm^2^, 2.5%) in combination with a 525/50 emission mirror was employed to image the green channel. A 559‐nm laser line (120 mW/cm^2^, 2.0%) and a 643/50 emission filter was used for red channel recordings. For monitoring changes of [Ca^2+^]_i_ in response to external stimuli, cells were incubated with the acetoxymethyl ester of the Ca^2+^ indicator Fluo‐4 (Life Technologies), 5 µM in DMEM (1 g/L glucose) for 25 min at 37°C and 5% CO_2_. The frame time was set to 3.9 s, with images acquired in 4 s intervals. For imaging, MIN6 cells were grown as pseudoislets of ~70% confluence. Imaging experiments were performed in HEPES buffer (imaging buffer; in mM: 115 NaCl, 1.2 CaCl_2_, 1.2 MgCl_2_, 1.2 K_2_HPO_4_, and 20 HEPES, pH 7.4). Washing was performed on adherent cells in LabTek imaging dishes. For this, the growth medium was removed and cells were gently washed 3× with pre‐warmed imaging buffer at respective glucose concentrations to remove extracellular signaling factors. For imaging, cells were placed in 150 µl pre‐warmed imaging buffer at indicated glucose concentrations.

### 2.9 Analysis of imaging data

The open access software tool Fiji (Schindelin et al., 2012) was applied for the extraction of fluorescence intensities from individual cells. Intensities were calculated relative to the maximum detected fluorescence intensity (*F*/*F*_0_). Representative traces of cells within a field of view were averaged or numbers of detected high‐intensity [Ca^2+^]_i_ events per 60 s interval were determined. The height of each [Ca^2+^]_i_ event was determined relative to the highest detected peak per trace. This served as a criterion to group [Ca^2+^]_i_ oscillations into low‐intensity (<60% of highest peak) and high‐intensity (≥60% of highest peak) events. Per condition or tested stimulus, 4–6 independent experiments were performed under identical settings. At least 50 individual representative MIN6 were picked per condition and their responses were averaged. Therefore, the number of high‐intensity [Ca^2+^]_i_ oscillations refers to the sum of events from 50 representative cells within intervals of 60 s. OriginLab, version 8.5 was applied for statistical analysis and plotting.

## 3 RESULTS

### 3.1 ATP‐stimulated [Ca^2+^]_i_ oscillations and insulin secretion in pre‐washed MIN6 and mouse primary β‐cells

Static incubation of MIN6 cells in the presence of 11 mM glucose is characterized by periodic transients of the cytosolic Ca^2+^ concentration ([Ca^2+^]_i_ oscillations), with cell‐to‐cell variability in frequency and shape of oscillations. Using the Ca^2+^ sensitive cell‐permeant version of the fluorescent indicator Fluo‐4, we observed that MIN6 cells respond to increasing concentrations of external glucose with enhanced GSIS, along with differential levels of [Ca^2+^]_i_ oscillations (Figure S1) (Gee et al., 2000). This is in line with literature reports on MIN6 cells and isolated pancreatic islets (Ishihara et al., 1993). To quantitatively describe [Ca^2+^]_i_ events, traces of representative cells were averaged and counts for detected “high‐intensity [Ca^2+^]_i_ events” per 60 s interval were determined. Traces were normalized to the highest detected peak (intensity‐based normalization). Addition of KCl at the end of experiments caused cell depolarization and induced high‐intensity [Ca^2+^]_i_ transients with intensities that compared well to ATP‐mediated cell depolarization. Consequently, intensity‐based normalization to the highest detected intensity peaks did not change the overall appearance of traces (Figure S2). For comparison, exemplary traces without normalization are shown in Figure S3. Alternatively, comparison of cellular activity based on the area under the curve (AUC) of [Ca^2+^]_i_ traces was performed. This representation does not reflect observed cellular behavior and insulin secretion in response to external stimuli (Figure S4).

**FIGURE 1:**
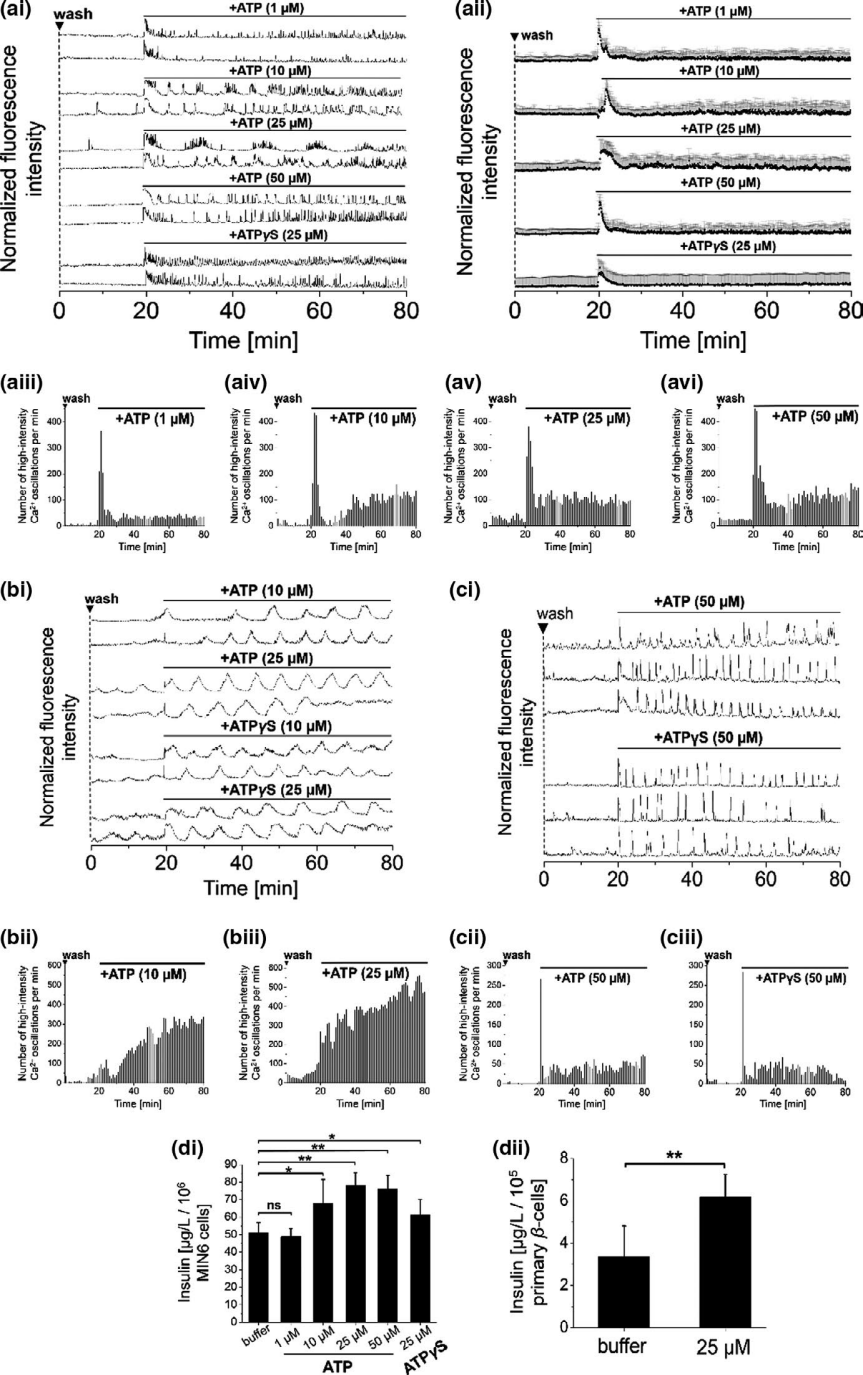
Addition of ATP‐stimulated [Ca^2+^]_i_ oscillations and insulin secretion in β‐cell lines and primary β‐cells. Addition of ATP or adenosine‐5′‐[γ‐thio]triphosphate (ATPγS) to pre‐washed (a) MIN6, (b) mouse primary β‐cells or (c) 1.1B4 cells immediately started [Ca^2+^]_i_ oscillations in a concentration‐dependent manner (concentrations as indicated). Representative single [Ca^2+^]_i_ traces of (ai) MIN6, (bi) mouse primary β‐cells and (ci) 1.1B4 cells, recorded with the Ca^2+^ indicator Fluo‐4. (aii) Averaged [Ca^2+^]_i_ traces from MIN6 cells. Numbers of detected high‐intensity [Ca^2+^]_i_ events per 60 s interval from (aiii‐vi) MIN6, (bii+iii) mouse primary β‐cells, and (cii+iii) 1.1B4 cells. (d) Insulin secretion, as determined from (di) MIN6 and (dii) mouse primary β‐cells in the presence of ATP or ATPγS. For [Ca^2+^]_i_ data, averages of *n* = 50 MIN6, 1.1B4 and *n* = 30 mouse primary β‐cells are shown. Imaging experiments were performed in the presence of 11 mM glucose (MIN6 cells and 1.1B4 cells) and 5 mM glucose (mouse primary β‐cells). Washes are indicated by ▼. Insulin measurements were performed in quadruplicate (ANOVA. **p < 0.01, ns = not significant = *p* > 0.05, with repeated measures as necessary). Error bars present SD

ATP has been previously identified among para‐ and autocrine signaling factors in the extracellular space of rodent and human pancreatic islets to act on P2 purinergic receptors (Jacques‐Silva et al., 2010; Tengholm, 2014). Effects of autocrine ATP as an extracellular signaling factor were investigated on the pancreatic cell lines MIN6 (murine) (Ishihara et al., 1993) and 1.1B4 (human) (McCluskey et al., 2011) as well as on isolated mouse primary β‐cells. Initial washing of MIN6, 1.1B4, and mouse primary β‐cells was intended to remove extracellular (autocrine) signaling factors, including ATP and FAs. Pre‐washed cells showed significantly reduced [Ca^2+^]_i_ oscillations (Figure S5), that spontaneously recovered over time, as the extracellular signaling network was restored (Hauke et al., 2018). To determine immediate effects of purinergic receptor activation on [Ca^2+^]_i_ oscillations and insulin secretion, pre‐washed cells were treated with ATP at concentration ranges of 1–50 µM. Notably, following the removal of extracellular autocrine signaling factors in a prewashing step, addition of ATP was sufficient to instantaneously induce intermittent [Ca^2+^]_i_ oscillations in a concentration‐dependent manner. Similar thresholds were observed for pre‐washed MIN6 and mouse primary β‐cells. Whereas ATP only induced single prominent [Ca^2+^]_i_ spikes at low concentrations (1 µM, Figure 1ai+iii), continuous [Ca^2+^]_i_ oscillations were evoked at higher ATP concentrations (10–25 µM) in pre‐washed MIN6 and mouse primary β‐cells, as indicated by counts of high‐intensity [Ca^2+^]_i_ oscillations (Figure 1 aiv‐v, bii+iii). 1.1B4 cells were stimulated by ATP only at concentrations of 50 µM or higher (Figure 1c). Concentrations beyond 50 µM did not further potentiate [Ca^2+^]_i_ oscillations or increase insulin secretion. Extracellular ATP was expected to be hydrolyzed by membrane‐localized ectonucleotidases. Therefore, we applied the non‐hydrolyzable ATP‐derivative adenosine‐5´‐[γ‐thio]triphosphate (ATPγS) to pre‐washed MIN6 cells to test the immediate effects of ATP on [Ca^2+^]_i_ oscillations and to exclude effects from ATP hydrolytic products. ATPγS potently induced [Ca^2+^]_i_ oscillations already at 25 µM in pre‐washed MIN6 and mouse primary β‐cells (Figure 1a+b) and also stimulated 1.1B4 cells at 50 µM (Figure 1c). Insulin secretion from MIN6 cells was stimulated up to ~1.6‐fold by ATP (10–50 µM) and ~1.4‐fold by ATPγS (25 µM) over buffer levels (Figure 1di). This data are also shown in dot‐plot representation in Figure S8. Insulin release from mouse primary β‐cells was potentiated ~1.5‐fold by ATP at 25 µM (Figure 1dii). Experiments on mouse primary β‐cells were performed in the presence of 5 mM glucose with more than 90% of cells responding to the ATP stimulus following washing. Cells showed low frequency [Ca^2+^]_i_ oscillations (Figure 1bi), as described by literature (Tengholm & Gylfe, 2009). However, in the presence of 11 mM glucose, mouse primary β‐cells showed high‐ high‐frequency [Ca^2+^]_i_ oscillations (Figure S6). Whereas full stimulation of MIN6 cells by ATP was demonstrated in the presence of 11 mM glucose, addition of ATP to MIN6 cells in the presence of 3 mM glucose evoked single pronounced [Ca^2+^]_i_ transients (Figure S7), in line with literature reports (Jacques‐Silva et al., 2010).

### 3.2 Depletion of extracellular ATP levels by recombinant apyrase regulated [Ca^2+^]_i_ oscillations

**FIGURE 2:**
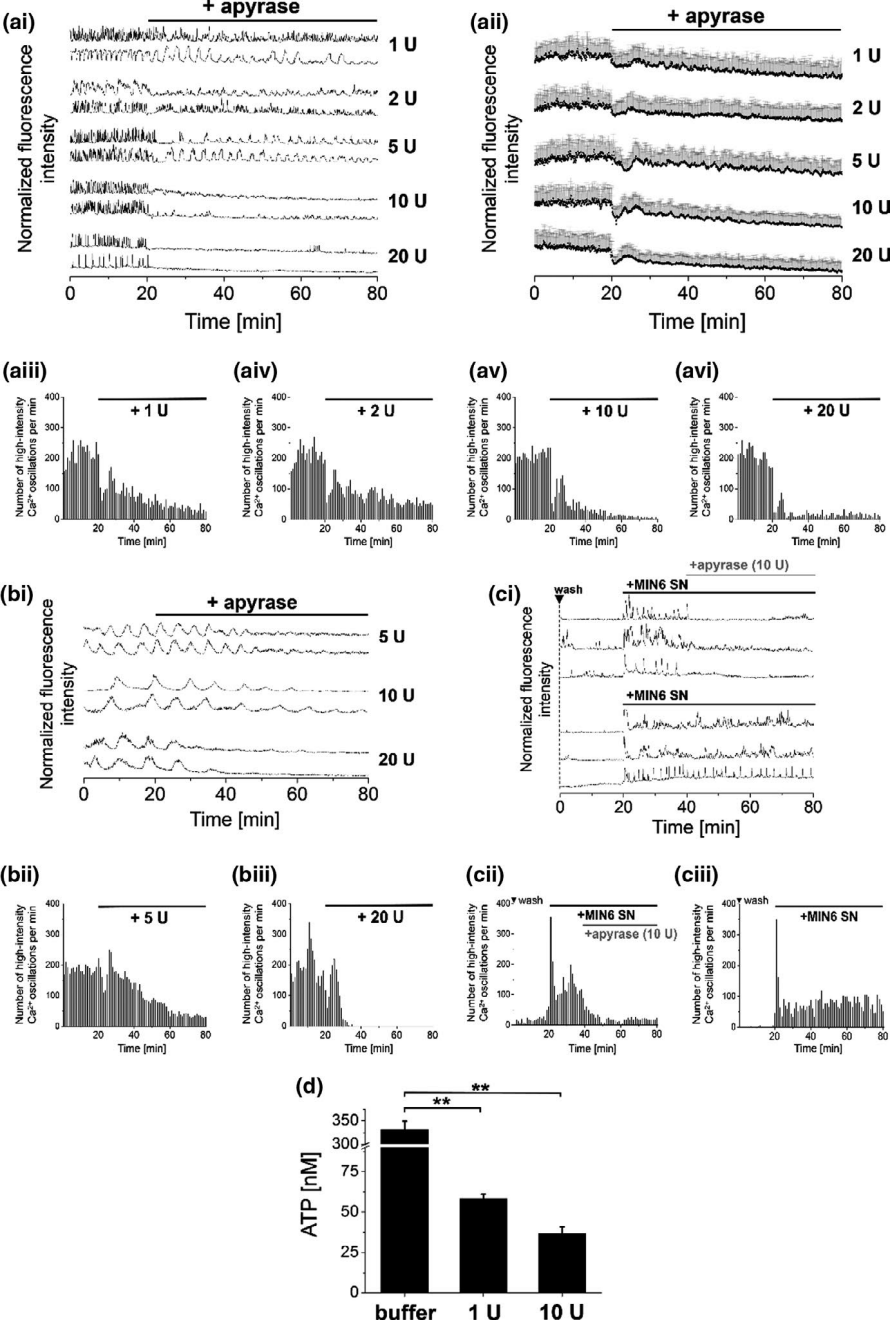
Addition of recombinant apyrase to glucose‐stimulated β‐cell lines and primary β‐cells reduced levels of endogenous ATP and [Ca^2+^]_i_ oscillations. Addition of recombinant apyrase to [Ca^2+^]_i_ oscillating (a) MIN6, (b) mouse primary β‐cells, and (c) 1.1B4 cells reduced [Ca^2+^]_i_ oscillations in an activity‐dependent manner (applied enzyme activity in units (U) as indicated). Representative single [Ca^2+^]_i_ traces of (ai) MIN6, (bi) mouse primary β‐cells, and (ci) 1.1B4 cells, recorded with the Ca^2+^ indicator Fluo‐4. (aii) Averaged [Ca^2+^]_i_ traces from MIN6 cells. (c) Application of supernatant that was preloaded on MIN6 cells started [Ca^2+^]_i_ oscillations in 1.1B4 cells. Addition of recombinant apyrase (10 U) reduced [Ca^2+^]_i_ oscillations that were stimulated by MIN6 supernatant. (aiii‐vi, bii+iii, cii+iii) Numbers of detected high‐intensity [Ca^2+^]_i_ events per 60 s interval. (d) Levels of endogenous ATP in the supernatant of MIN6 cells as determined in the presence of recombinant apyrase in different activities. Imaging was performed in the presence of 11 mM glucose (MIN6 cells and 1.1B4 cells) and 5 mM glucose (mouse primary β‐cells). Shown are averages of *n* = 50 MIN6 or 1.1B4 and *n* = 30 mouse primary β‐cells. Washes are indicated by ▼. ATP measurements were performed in quadruplicate (ANOVA. **p < 0.01, ns = not significant = *p* > 0.05, with repeated measures as necessary). Error bars present SD

Extracellular ATP pools inside islets of Langerhans are dynamically regulated by the exocytosis of ATP from nucleotide‐filled vesicles and by membrane‐localized ecto‐ATPases (such as apyrase) that convert ATP to adenosine (Lavoie et al., 2010). Therefore, ecto‐ATPases are crucial regulators for the duration and extent of purinergic signaling (Bours et al., 2006). Accordingly, we found that addition of a recombinant ATP‐diphosphohydrolase (apyrase, 1–20 U) to glucose‐stimulated MIN6 and mouse primary β‐cells reduced and eventually stopped [Ca^2+^]_i_ oscillations in an activity‐dependent manner (Figure 2a+b). As recombinant apyrase action was restricted to the extracellular space, we suspected that extracellular pools of ATP were selectively depleted. Whereas addition of apyrase to MIN6 cells at activities of 1–5 U lowered but did not abolish [Ca^2+^]_i_ oscillations (Figure 2ai‐iii), [Ca^2+^]_i_ oscillations were immediately stopped in the presence of 10 U or higher activities (Figure 2ai+v+vi). Based on a luciferase‐based assay, we determined ATP levels in the supernatant of glucose‐stimulated MIN6 cells in the nM range (Figure 2d, buffer). This data are shown in dot‐plot representation in Figure S8. Extracellular ATP levels inversely correlated with the applied apyrase activity in the presence of 11 mM glucose (Figure 2d). Comparable effects on [Ca^2+^]_i_ oscillations were observed for primary mouse β‐cells upon addition of recombinant apyrase (Figure 2b), indicating the essential and universal role of ATP for β‐cell activity.

1.1B4 cells did not show spontaneous [Ca^2+^]_i_ oscillations even in the presence of elevated glucose levels. However, supernatant that was preincubated on MIN6 cells for 1.5 h at 37°C instantaneously evoked continuous [Ca^2+^]_i_ oscillations when added to 1.1B4 cells. [Ca^2+^]_i_ oscillations were immediately reduced by apyrase‐mediated reduction of ATP levels within the supernatant. This again indicated the essential role of individual soluble autocrine signaling factors for β‐cell activity. Addition of recombinant apyrase (10 U) also reduced and stopped [Ca^2+^]_i_ oscillations in pre‐stimulated 1.1B4 cells (Figure 2c). Therefore, selective depletion of ATP from the entire spectrum of autocrine factors in the supernatant was sufficient to reduce [Ca^2+^]_i_ oscillations.

### 3.3 Selective inhibition of cellular ectonucleotidases increased levels of extracellular ATP and stimulated [Ca^2+^]_i_ oscillations as well as insulin secretion

**FIGURE 3:**
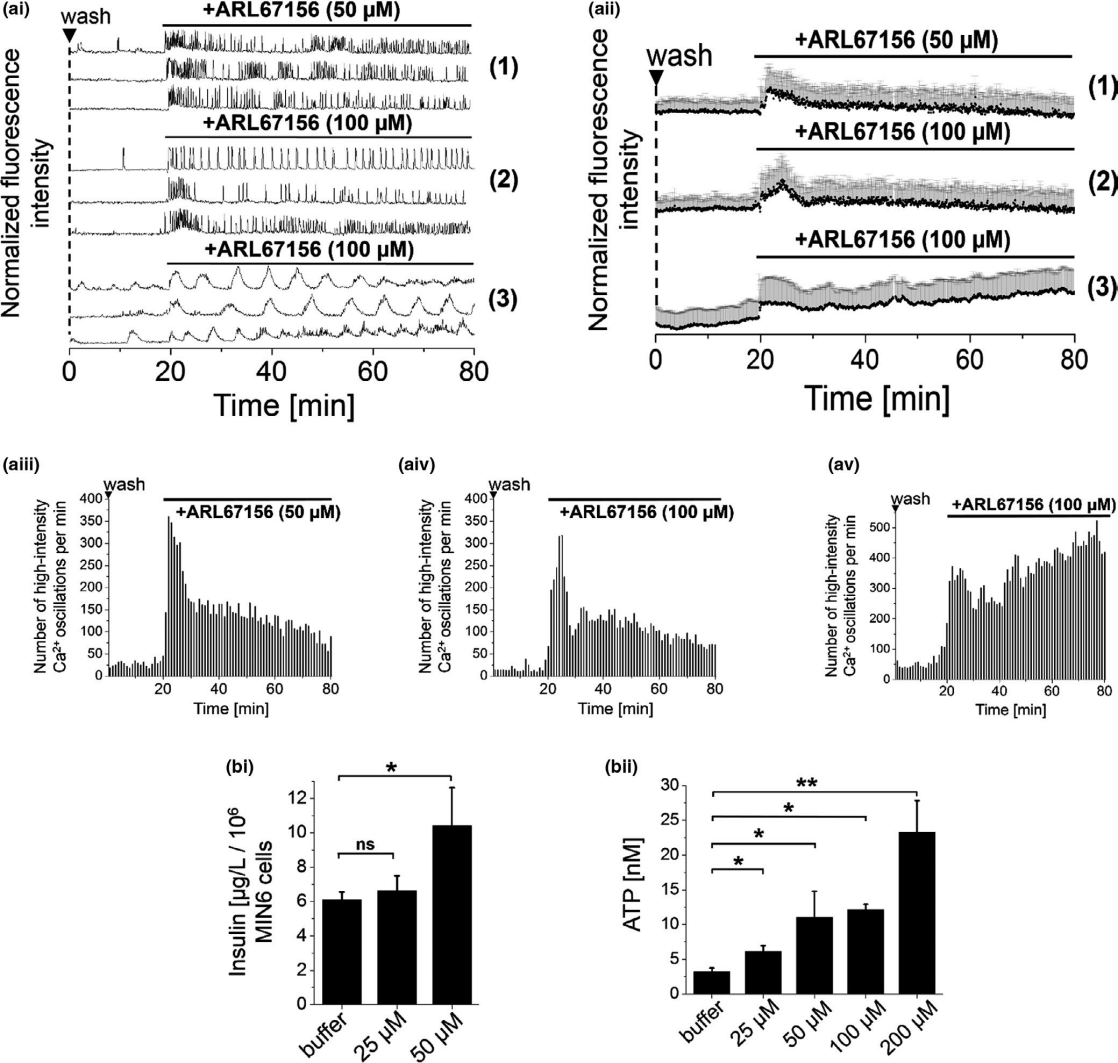
Inhibition of endogenous apyrase increased extracellular levels of ATP and stimulated [Ca^2+^]_i_ oscillations and insulin secretion. (a) Addition of the selective ecto‐ATP‐nucleotidase inhibitor ARL67156 (50 µM) to pre‐washed (1 + 2) MIN6 and (Bours et al., 2006) mouse primary β‐cells started [Ca^2+^]_i_ oscillations (concentrations as indicated). Representative (ai) single and (aii) averaged [Ca^2+^]_i_ traces, recorded with the Ca^2+^ indicator Fluo‐4. Numbers of detected high‐intensity [Ca^2+^]_i_ events per 60 s interval in (aiii+iv) MIN6 and (av) mouse primary β‐cells. (bi) Insulin and (bii) ATP levels, as determined from the supernatant of MIN6 cells in the presence of ARL67156 in different concentrations. Shown are averages of *n* = 50 MIN6 and *n* = 30 mouse primary β‐cells. Imaging was performed in the presence of 11 mM glucose (MIN6 cells) and 5 mM glucose (mouse primary β‐cells). Washes are indicated by ▼. Insulin and ATP measurements were performed in quadruplicate (ANOVA. *p < 0.05, **p < 0.01, ns = not significant = *p* > 0.05, with repeated measures as necessary). Error bars present SD

Secreted ATP is rapidly cleared by cellular ecto‐ATPases, localized on the outer leaflet of plasma membranes (Burnstock & Novak, 2013). Hydrolyzing ATP to adenosine, ecto‐ATPases have been reported to act as essential determinants that regulate the extent and the duration of purinergic signaling within pancreatic islets (Bours et al., 2006). Therefore, we aimed at manipulating levels of extracellular ATP and thereby [Ca^2+^]_i_ oscillations and insulin secretion by the selective inhibition of endogenous ectonucleotidases. We found that application of the selective apyrase inhibitor ARL67156 (50 µM) (Lévesque et al., 2007) to pre‐washed MIN6 cells immediately induced [Ca^2+^]_i_ oscillations (Figure 3a (1+2), along with increased ATP levels and insulin release (Figure 3b). This revealed fast enzymatic turnover rates of endogenously released ATP in MIN6 cells under normal conditions. Comparable effects of ecto‐ATPase inhibition were observed on [Ca^2+^]_i_ oscillations in mouse primary β‐cells (Figure 3a, Bours et al., 2006). Increasing the ARL67156 concentration to 100 µM did not further potentiate [Ca^2+^]_i_ oscillations (Figure 3a) or increase ATP and insulin levels (Figure 3b), respectively. This insulin data are also shown in dot‐plot representation in Figure S8.

### 3.4 Manipulation of [Ca^2+^]_i_ oscillations by selective enzymatic depletion and replenishment of extracellular ATP levels

**FIGURE 4:**
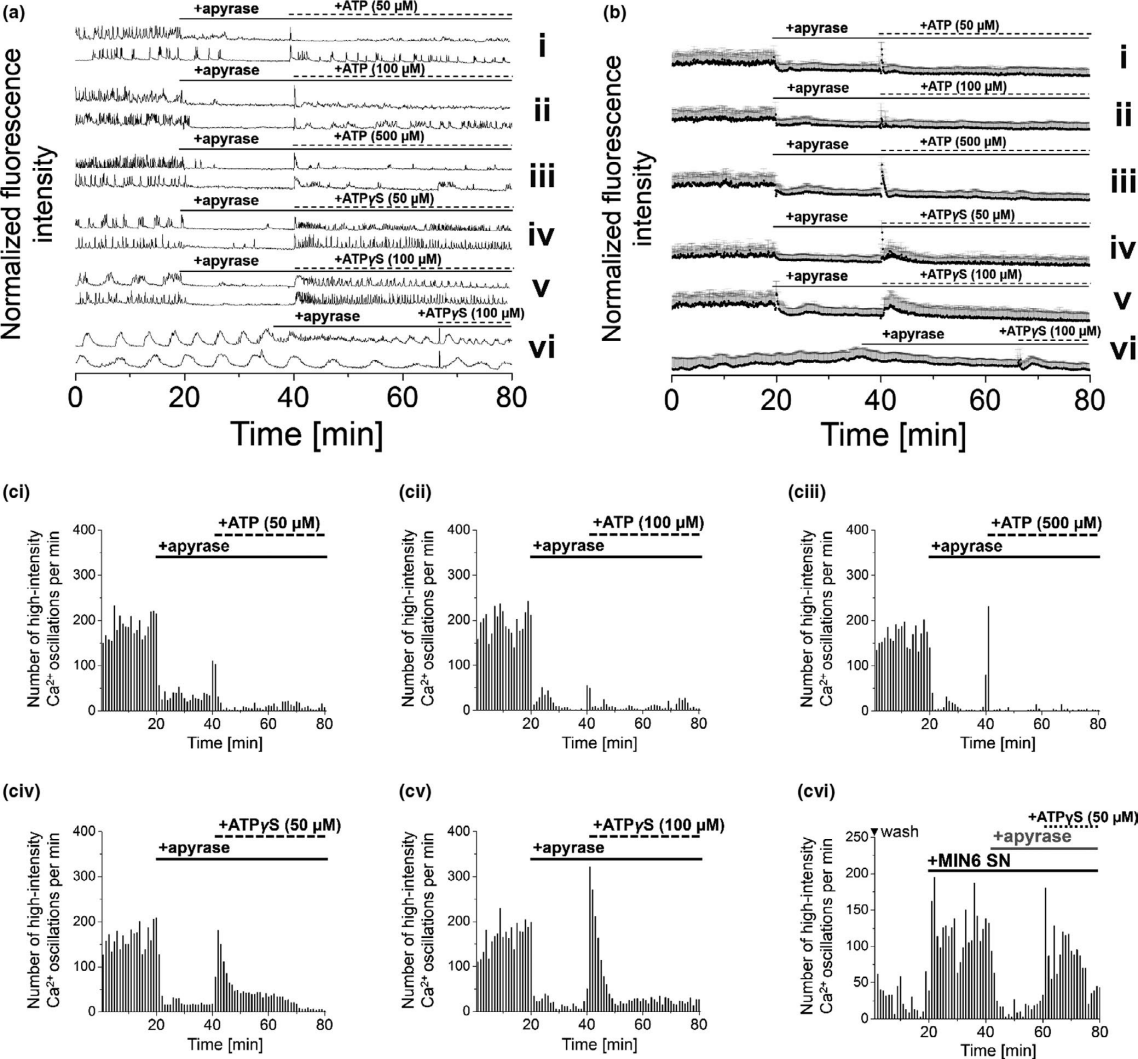
Modulation of [Ca^2+^]_i_ oscillations by selective enzymatic depletion and replenishment of extracellular ATP. Application of recombinant apyrase (10 U) to [Ca^2+^]_i_ oscillating MIN6 cells reduced [Ca^2+^]_i_ oscillations, which were recovered by the addition of ATP or the stable ATP‐analogue ATPγS (concentrations as indicated). (ai) Representative single and (aii) average [Ca^2+^]_i_ traces from MIN6 cells and mouse primary β‐cells (indicated by asterisk), recorded with the Ca^2+^ indicator Fluo‐4. Numbers of detected high‐intensity [Ca^2+^]_i_ events per 60 s interval recorded from (aiii‐vii) MIN6 or (aviii) 1.1B4 cells. Imaging was performed in the presence of 11 mM glucose (MIN6 and 1.1B4 cells) and 5 mM glucose (mouse primary β‐cells). Shown are averages of *n* = 50 MIN6, 1.1B4, and *n* = 30 mouse primary β‐cells. Washes are indicated by ▼

Based on these findings, we concluded that [Ca^2+^]_i_ oscillations essentially rely on levels of extracellular ATP. Therefore, we suspected that [Ca^2+^]_i_ oscillations can be modulated by enzymatic depletion and replenishment of extracellular ATP levels. To test this hypothesis, recombinant apyrase (10 U) was added to glucose‐stimulated oscillating MIN6 cells to efficiently reduce [Ca^2+^]_i_ oscillations, as shown before (Figure 2a). The immediate stimulatory potency of ATP, applied to apyrase‐treated MIN6 cells, was restricted to firing a single [Ca^2+^]_i_ transient, but did not re‐start continuous [Ca^2+^]_i_ oscillations, even at high concentrations of 100–500 µM (Figure 4a–c i‐iii). Following the supplier, 10 U of recombinant apyrase hydrolyze 10 µmol ATP per minute. Accordingly, ATP added at 50–500 µM final concentrations was supposed to be hydrolyzed within less than 0.5 s. To avoid effects from enzymatic degradation of ATP, we added the non‐hydrolyzable ATP‐derivative ATP*γ*S to apyrase‐treated MIN6 cells. ATP*γ*S applied at 50 µM or 100 µM final concentrations restarted spontaneously occurring and continuous [Ca^2+^]_i_ transients, even in the presence of apyrase (Figure 4a‐c iv+v). Comparable effects of ATP*γ*S were observed for mouse primary β‐cells (Figure 4a+b vi) and for 1.1B4 cells, following stimulation by MIN6 supernatant (Figure 4c vi).

### 3.5 ATP stimulates cellular activity acting on purinergic receptors

**FIGURE 5:**
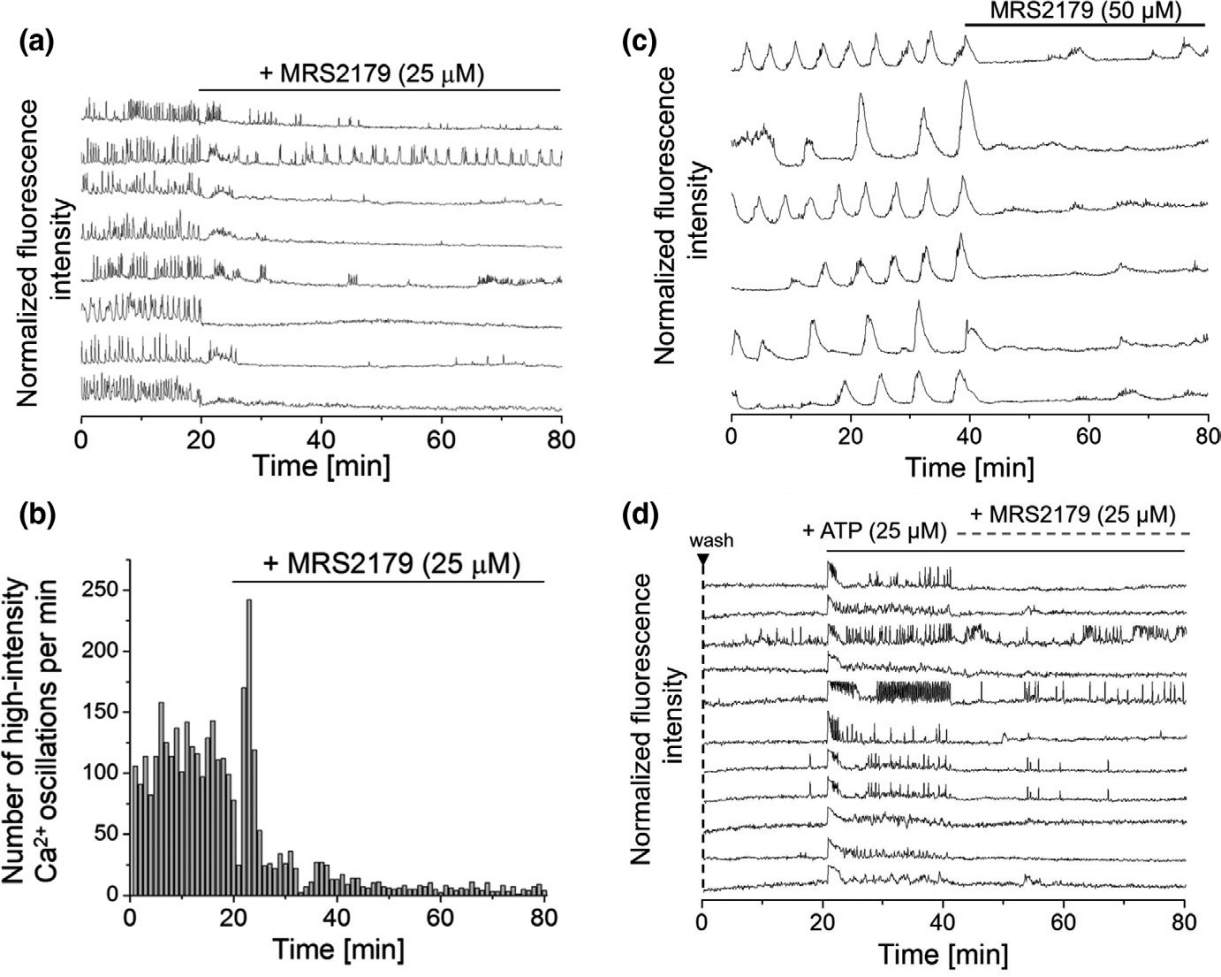
Modulation of [Ca^2+^]_i_ oscillations in MIN6 cells by ATP and the P2Y antagonist MRS2179 unravels a possible mechanism of ATP action. (a) Addition of the P2Y antagonist MRS2179 (25 µM) to [Ca^2+^]_i_ oscillating MIN6 cells reduced and finally stopped [Ca^2+^]_i_ oscillations, indicating the essential role of ATP‐mediated P2Y stimulation for cell activity. (b) Treatment of MIN6 cells with MRS2179 (25 µM)––corresponding numbers of detected high‐intensity [Ca^2+^]_i_ events per 60 s interval. Shown are averages acquired from *n* = 50 MIN6 cells. (c) Addition of MRS2179 (50 µM) to mouse primary β‐cells reduced and stopped [Ca^2+^]_i_ oscillations. Findings on primary β‐cells compared well to observations on MIN6 cells. (d) Combined modulation of [Ca^2+^]_i_ oscillations by ATP and MRS2179. Addition of ATP (25 µM) to pre‐washed MIN6 cells‐stimulated [Ca^2+^]_i_ oscillations that were reduced and partly stopped by the P2Y antagonist MRS2179 (25 µM), probably by out‐competition of ATP from the receptor. Imaging was performed in the presence of 11 mM glucose for MIN6 cells and 5 mM glucose for mouse primary β‐cells

To clarify the mechanism underlying ATP‐mediated stimulation of [Ca^2+^]_i_ oscillations and insulin secretion, MIN6 cells and mouse primary β‐cells were treated with the P2Y‐receptor antagonist MRS2179 (Salehi et al., 2005). Addition of MRS2179 to [Ca^2+^]_i_ oscillating MIN6 cells or mouse primary β‐cells in the presence of 5 mM or 11 mM glucose, respectively, reduced and stopped [Ca^2+^]_i_ oscillations, indicating the essential role of this receptor for cellular activity (Figure 5a–c and Figure S6).

To demonstrate the reversibility of P2Y stimulation, pre‐washed MIN6 cells were first stimulated by ATP (25 µM) to evoke continuous [Ca^2+^]_i_ oscillations. Subsequent addition of MRS2179 (25 µM) to pre‐stimulated cells reduced and partially stopped ATP‐induced [Ca^2+^]_i_ oscillations (Figure 5d), indicating the essential role of P2Y receptor stimulation for cellular activity and ATP action.

### 3.6 Addition of oleic acid recovered [Ca^2+^]_i_ oscillations in MIN6 cells in the presence of apyrase

**FIGURE 6:**
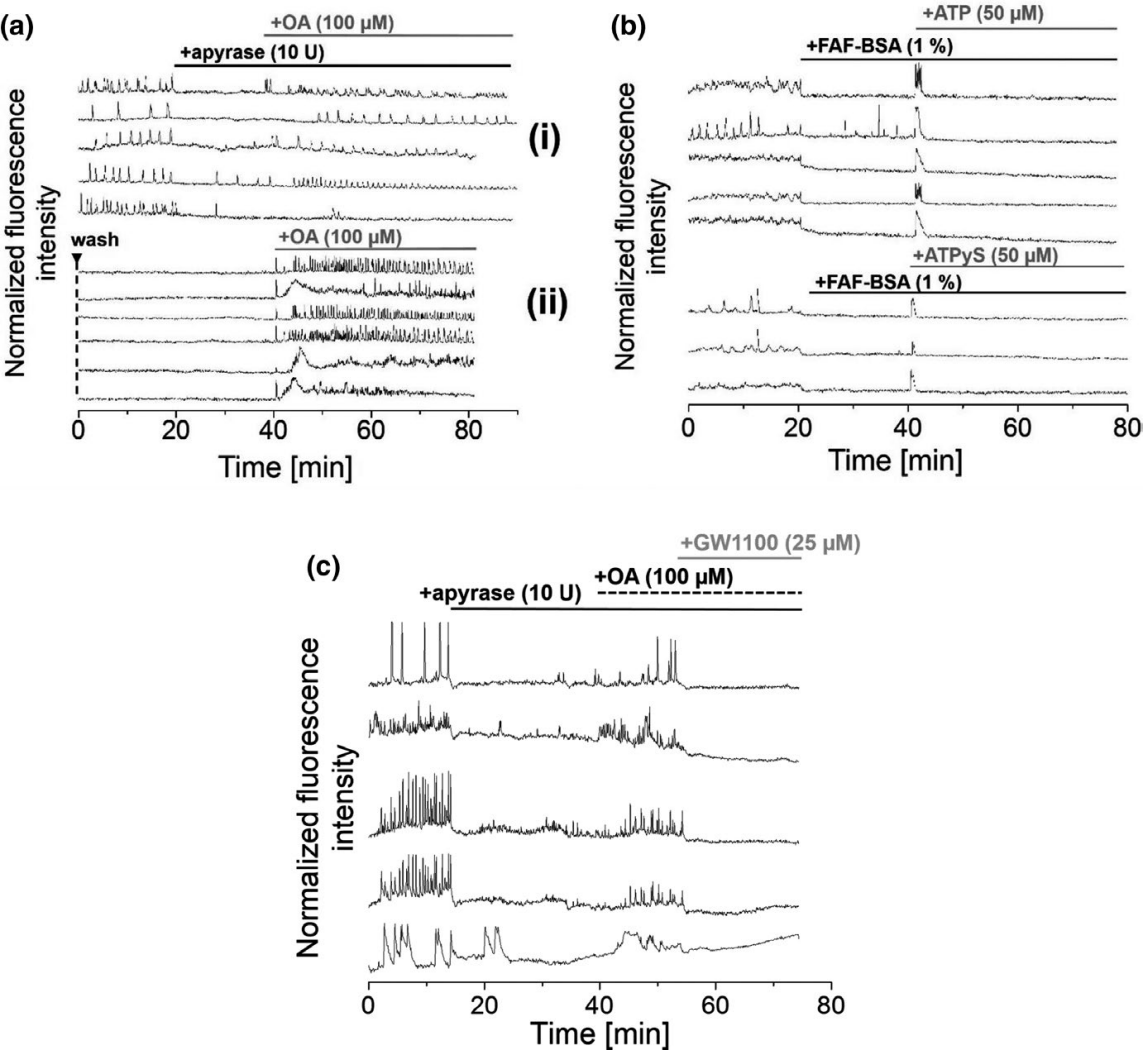
Interplay of different types of extracellular autocrine signaling factors regulates the activity of MIN6 cells (ai) [Ca^2+^]_i_ oscillations in glucose‐stimulated MIN6 cells stopped in the presence of recombinant apyrase (10 U), to recover upon addition of oleic acid (100 µM). (aii) As a control, pre‐washed MIN6 cells were treated with oleic acid (100 µM), which evoked even stronger responses in [Ca^2+^]_i_ oscillations. (b) Addition of ATP or ATPγS (50 µM) to FAF‐BSA‐ treated MIN6 cells yielded a single high‐intensity [Ca^2+^]_i_ transient but did not evoke spontaneously occurring continuous [Ca^2+^]_i_ oscillations. (c) Addition of recombinant apyrase (10 U) reduced and stopped [Ca^2+^]_i_ oscillations in MIN6 cells. Subsequent application of oleic acid (100 µM) partly resumed [Ca^2+^]_i_ oscillations which was finally stopped by the addition of the GPR40 antagonist GW1100 (25 µM), pointing to an interplay of various autocrine extracellular signaling factors that regulate cell activity. Imaging was performed in the presence of 11 mM glucose. Washes are indicated by ▼

In our previous work, we demonstrated that addition of FA‐free (FAF‐)BSA to glucose‐stimulated MIN6 and mouse primary β‐cells immediately reduced and stopped [Ca^2+^]_i_ oscillations and insulin secretion. Here, we already reported the essential role of autocrine signaling factors for [Ca^2+^]_i_ oscillations and insulin secretion (Hauke et al., 2018). Following the herein presented observations of the essential role of ATP for [Ca^2+^]_i_ oscillations and insulin secretion, we were interested in whether different autocrine signaling factors can mutually compensate the loss of others. For this, ATP levels were depleted by the addition of recombinant apyrase (10 U) to reduce and stop [Ca^2+^]_i_ oscillations in glucose‐stimulated MIN6 cells. Addition of oleic acid (OA) to apyrase‐treated MIN6 cells restarted [Ca^2+^]_i_ oscillations (Figure 6ai), indicating that FAs may compensate for the loss of ATP. As a control, pre‐washed MIN6 cells were treated with OA in the absence of apyrase. This induced even stronger recovery of [Ca^2+^]_i_ oscillations (Figure 6aii). Notably, following the removal of FAs by FAF‐BSA (1%), addition of ATP or ATP*γ*S (50 µM, each) just evoked a single pronounced [Ca^2+^]_i_ transient without continuous [Ca^2+^]_i_ oscillations (Figure 6b).

To demonstrate the interplay of different types of extracellular autocrine signaling factors and their role for the regulation of cellular activity, extracellular ATP pools were first depleted by the addition of recombinant apyrase (10 U). Subsequent addition of OA (100 µM) to MIN6 cells partially restarted [Ca^2+^]_i_ oscillations, which were reduced and stopped by the application of the GPR40 antagonist GW1100 (25 µM) (Figure 6c) (Briscoe et al., 2006). This indicated the essentiality of a balanced interplay of different types of autocrine signaling factors for cellular activity and the role of GPR40 for the recovery of [Ca^2+^]_i_ oscillations following OA treatment.

## 4 DISCUSSION

The role of soluble autocrine signaling molecules is increasingly recognized as a crucial factor for β‐cell synchronization and pulsatile insulin secretion. Previous work demonstrated that populations of β‐cells are stimulated by extracellular ATP, inducing [Ca^2+^]_i_ transients. Further, it has been shown that this message is transmittable to neighboring cells via intermittent release of ATP without direct physical contact (Hellman et al., 2004). Therefore, ATP‐mediated synchronization of the β‐cell secretory machinery is a prime example for effects that autocrine signaling factors have on insulin secretion (Grapengiesser et al., 2004, 2005; Salehi et al., 2005). Notably, electron micrographs show very narrow extracellular clefts between β‐cells that are just a few nanometers wide (Longnecker & Wilson, 1991). This confined extracellular space within pancreatic islets guarantees high dynamics, excellent molecule economy, and very rapid responses to external stimuli (Braun et al., 2012). Secretion or enzymatic turnover of relatively small amounts of endogenous (autocrine) signaling factors within the extracellular space result in significant concentration changes in the micromolar range. Following literature reports, ATP is released from insulin‐filled granules (Hutton et al., 1983; MacDonald et al., 2006; Wayne Leitner et al., 1975). Therefore, we were not surprised to find a direct correlation between ATP and insulin levels in the supernatant of MIN6 cells at different glucose concentrations. Monitoring [Ca^2+^]_i_ oscillations provided a direct, sensitive read‐out of rapid, transient individual β‐cell responses that would have been difficult to detect solely based on insulin ELISA bulk assays.

In our previous work, we reported that selective depletion of extracellular factors was sufficient to significantly reduce or stop [Ca^2+^]_i_ oscillations and insulin secretion in MIN6 and mouse primary β‐cells. Specifically, this was demonstrated by albumin‐mediated withdrawal of FAs from glucose‐stimulated MIN6 cells (Hauke et al., 2018). In the present work, we showed that extracellular ATP levels are a requirement for β‐cell activity and insulin secretion. In support for the hypothesis of an essential role of autocrine ATP levels for β‐cell activity (Hauke et al., 2018), our findings demonstrate that [Ca^2+^]_i_ oscillations and insulin secretion were directly modulated by the reduction and replenishment of extracellular ATP levels. Degrading ATP by apyrase significantly reduced extracellular ATP levels and stopped [Ca^2+^]_i_ oscillations. This was a surprising finding as other extracellular stimulating factors such as fatty acids were not removed.

Addition of ATP to pre‐washed MIN6 cells evoked continuous and spontaneously occurring [Ca^2+^]_i_ oscillations only in the presence of stimulating glucose levels (11 mM), but evoked only single [Ca^2+^]_i_ transients in the presence of sub‐stimulatory glucose levels (3 mM), which is in line with previous reports (Jacques‐Silva et al., 2010).

Experiments on mouse primary β‐cells were performed in the presence of 5 mM glucose. The activity curve of glucokinase shows the steepest increase at ~3–7 mM glucose resulting in very sensitive cellular behavior within this concentration range (Henquin et al., 2006; Salehi et al., 2006). Such glucose dependence is also consistent with the finding that high *K*_m_ GLUT2 is the dominating glucose transporter in rodent β‐cells (Tengholm & Gylfe, 2009). The observation that addition of 50 µM ARL67156 to MIN6 cells raises ATP levels just to nM scales, but at the same time significantly evokes insulin secretion suggests additional mechanisms of this antagonist, other than apyrase inhibition. This is also supported by the molecular structure of this inhibitor, strongly resembling ATP and therefore opening the potential for purinergic receptor interaction (Lévesque et al., 2007). Transfer of supernatant that was preloaded on MIN6 cells immediately started spontaneously occurring [Ca^2+^]_i_ oscillations in the human cell line 1.1B4. Even though 1.1B4 cells did not show spontaneous [Ca^2+^]_i_ oscillations in the presence of stimulatory glucose concentrations, addition of supernatant from pancreatic MIN6 cells‐induced [Ca^2+^]_i_ oscillations in 1.1B4 cells. This supported our hypothesis that autocrine signalling factors are released from pancreatic cells. Stimulation of 1.1B4 cells by buffer that was preincubated on a murine pancreatic cell line indicated the presence of universal autocrine signaling factors that are released into the medium by these secretory cell lines for mutual stimulation. Depletion of these [Ca^2+^]_i_ oscillations by the addition of recombinant apyrase indicated the essential role of ATP for the stimulation of 1.1B4 cells, as observed for murine MIN6 cells. Even though ATP signaling has been reported to show substantial species‐specific differences due to different subsets of purinergic receptors (Caicedo, 2013; Jacques‐Silva et al., 2010), our findings demonstrate the universal role of ATP as an essential factor for β‐cell activity and insulin secretion. Here, we demonstrate that ATP plays an important role in the extracellular auto‐modulatory (EAM) system by acting via the P2Y receptor family. Still, the question persists whether a lack of ATP can be compensated by other metabolites. Addition of FAs in the absence of ATP resumed [Ca^2+^]_i_ oscillations, indicating that other GPCRs, in this case the G_q_‐coupled GPR40, have the capability of compensating for the lack of a given autocrine factor, for instance if insulin secretion was stalled by longer periods of time. Such periods might be physiologically relevant under starving conditions when insulin and the concomitant ATP release is stalled for periods that permit full degrading of extracellular ATP levels by endogenous extracellular ATPases. This indicates that the EAM system has built‐in redundancies to prevent potential failure of the insulin secretion machinery. On the other hand, the supernatant of MIN6 cells was capable of fully reinstalling the EAM system in previously washed cells. Notably, responses in [Ca^2+^]_i_ oscillations that were induced by the addition of ATP to pre‐washed MIN6 cells were less pronounced compared to effects from preloaded buffer transfer (Hauke et al., 2018). Therefore, future work has to fully characterize the extracellular “secretome” of β‐cells. ATP, FAs, GABA, neuropeptide Y (NPY) as well as insulin itself likely constitute only a small fraction of the “cellular secretome.” Due to the small extracellular space within the islet, it seems likely that the levels of extracellular factors are locally controlled by metabolizing enzymes. Accordingly, we find a notable effect when using the ATPase inhibitor ARL67156, indicating that there is a constant release of ATP that is rapidly hydrolyzed under physiological conditions.

In summary, the release of ATP into the extracellular space of islets of Langerhans is a prime example of how the minute extracellular space is used as a local signaling compartment, not unlike a synapse. Similar to the synapse, metabolism of the signaling molecule is required to maintain the system in a dynamic state. In future work, it will be exciting and highly relevant to further identify other extracellular (autocrine) signaling molecules and to unravel their general role as part of a fundamental regulatory mechanism of β‐cell activity and insulin secretion.

## Supporting information



Supplementary Material
