# Supplementary material for: ATP is an essential autocrine factor for pancreatic β‐cell signaling and insulin secretion
Source: Physiol Rep. 2022 Jan 10;10(1):e15159. doi: 10.14814/phy2.15159 (PMC8743876; doi:10.14814/phy2.15159)
Supplement: Supplementary file 1 — Supplementary Material [file PHY2-10-e15159-s001.docx]

**Supplemental Data (SD)**

**ATP is an Essential Autocrine Factor for Pancreatic *β*-Cell Signaling and Insulin Secretion**

Sebastian Hauke^1^, Jona Rada^1^, Gergely Tihanyi^1^, Danny Schilling^1^ and Carsten Schultz^1,2*^

^1^ European Molecular Biology Laboratory (EMBL), Cell Biology & Biophysics Unit, Meyerhofstraße 1, 69117 Heidelberg, Germany.

^2^ Oregon Health & Science University (OHSU), L334, Department of Chemical Physiology and Biochemistry, 3181 SW Sam Jackson Park Road, Portland, OR 97239-3098, USA.

**
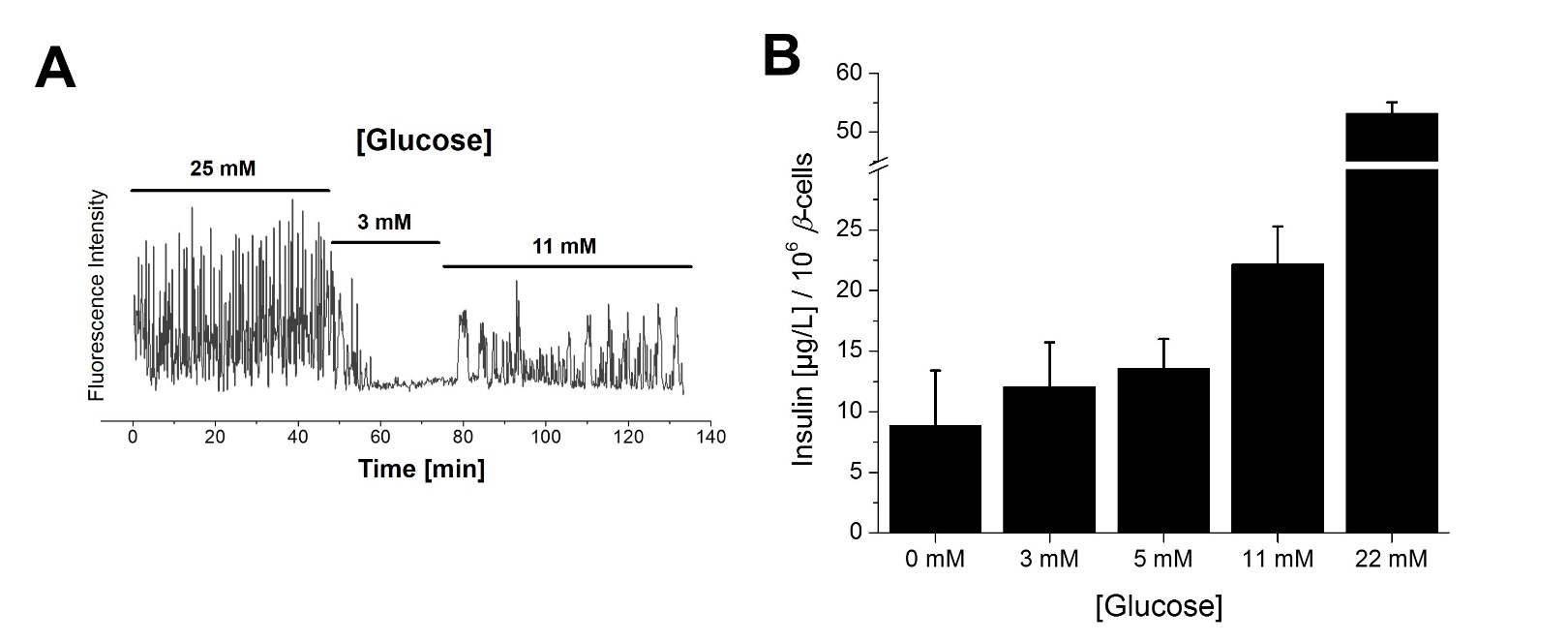
**

**Figure S1. Highly oscillatory Ca^2+^ responses to various levels of extracellular glucose.** Dependence of [Ca^2+^]_i_ oscillations in MIN6 cells and insulin secretion on the applied buffer glucose concentration. **(A)** In order to test the dynamics of the [Ca^2+^]_i_ response, MIN6 cells were treated with 3 mM, 11 mM and 25 mM glucose in buffer. No, intermediate or strong [Ca^2+^]_i_ oscillations were observed, respectively. For [Ca^2+^]_i_ imaging, the Ca^2+^ sensor R-GECO was transiently overexpressed in MIN6 cells. **(B)** Insulin levels at sub-stimulatory glucose concentrations (0 – 5 mM) were significantly lower compared to those observed at stimulatory glucose concentrations (11 and 22 mM). Experiments for the determination of insulin secretion were performed in quadruplicates.


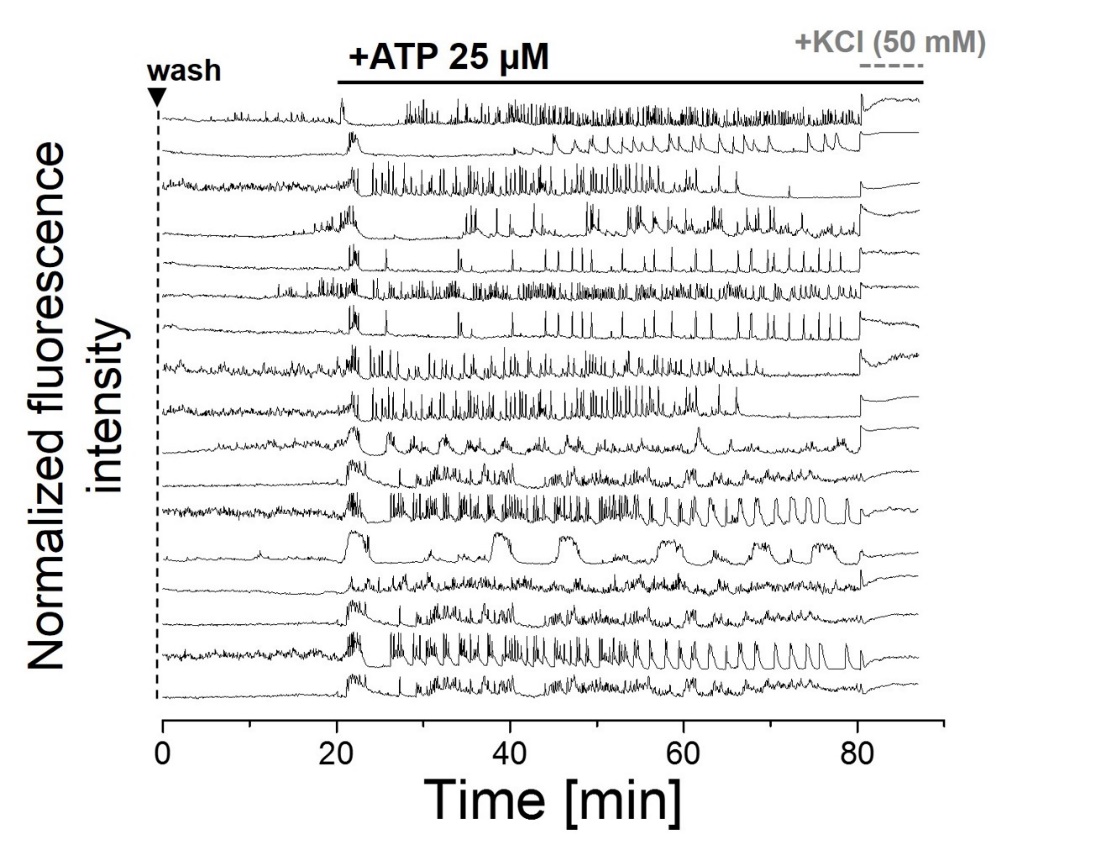


**Figure S2. Addition of ATP to pre-washed MIN6 cells immediately started [Ca^2+^]_i_ oscillations, followed by KCl-mediated depolarization of cells. Traces are normalized to the highest detected intensity.** Addition of ATP (25 µM) immediately started [Ca^2+^]_i_ oscillations in pre-washed MIN6 cells. Addition of KCl (50 mM) depolarized cells, along with a high-intensity spike at the end of the experiment. [Ca^2+^]_i_ oscillations immediately stopped after KCl-mediated depolarization of cells. Traces are normalized to highest detected intensity in each trace. Imaging was performed in the presence of 11 mM glucose.


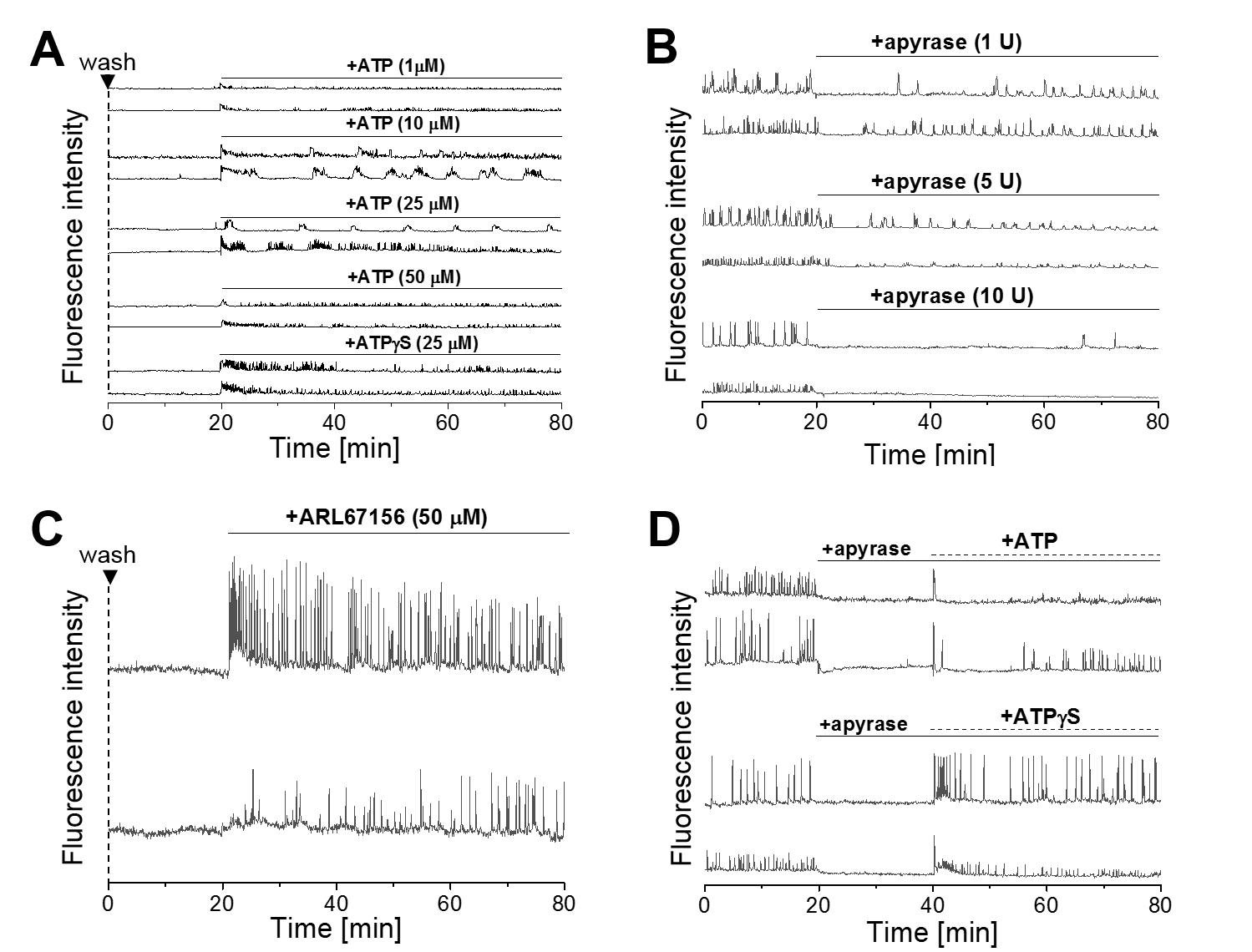


**Figure S3. Stimulation of MIN6 cells, represented as non-normalized [Ca^2+^]_i_ raw traces. (A)** Addition of ATP or adenosine-5´-[γ-thio]triphosphate (ATPγS) immediately started [Ca^2+^]_i_ oscillations in pre-washed MIN6 cells. This stimulatory effect was concentration dependent (concentrations as indicated). **(B)** Addition of recombinant apyrase to glucose-stimulated MIN6 cells reduced and finally stopped [Ca^2+^]_i_ oscillations (applied enzyme activity in units (U)). **(C)** Addition of the selective ecto-ATP-nucleotidase inhibitor ARL67156 (50 µM) to pre-washed MIN6 immediately started [Ca^2+^]_i_ oscillations. **(D)** Modulation of [Ca^2+^]_i_ oscillations by selective enzymatic depletion and replenishment of extracellular ATP levels. Application of recombinant apyrase (10 U) to MIN6 cells reduced [Ca^2+^]_i_ oscillations, which recovered by the addition of ATP or the stable ATP-analogue ATPγS (concentrations as indicated). Imaging was performed in the presence of 11 mM glucose.


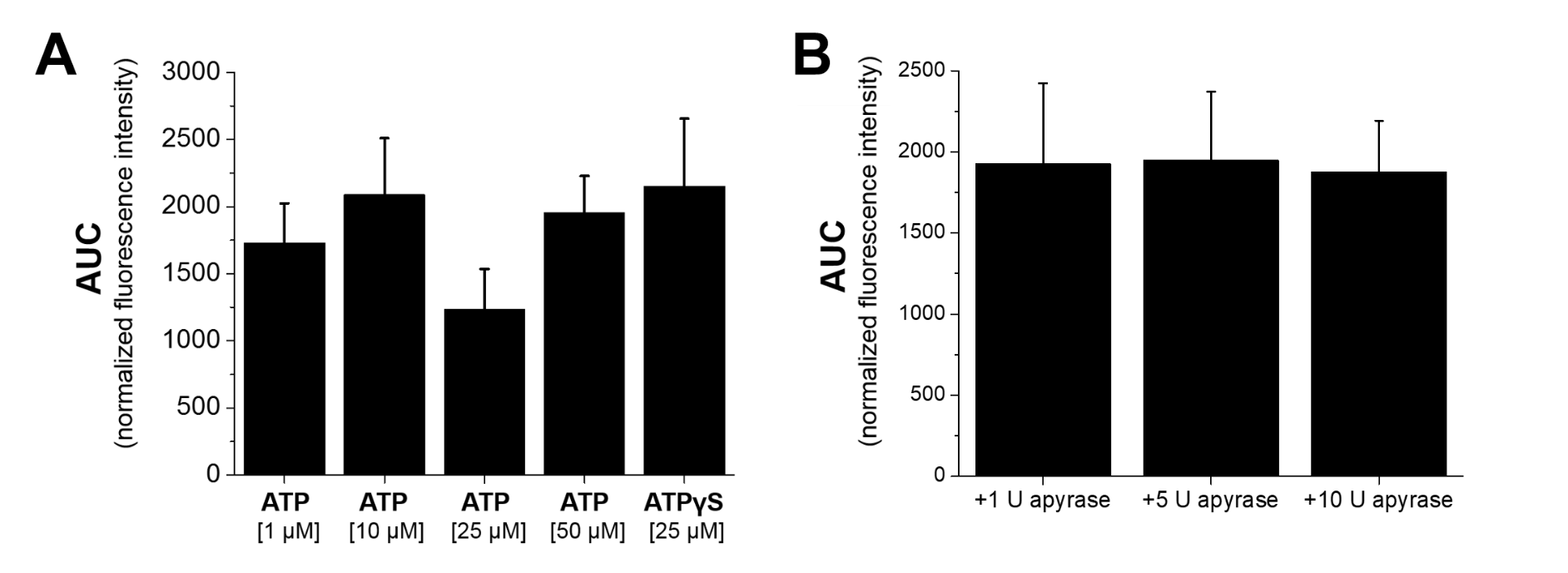


**Figure S4. Evaluation of the effects of ATP or apyrase on MIN6 cell activity, based on the area under the curve (AUC), as obtained from [Ca^2+^]_i_ recordings. (A)** Stimulation of MIN6 cells by ATP (concentrations as indicated). **(B)** Treatment of MIN6 cells with recombinant apyrase. Applied activities are indicated. Imaging was performed in the presence of 11 mM glucose.

**
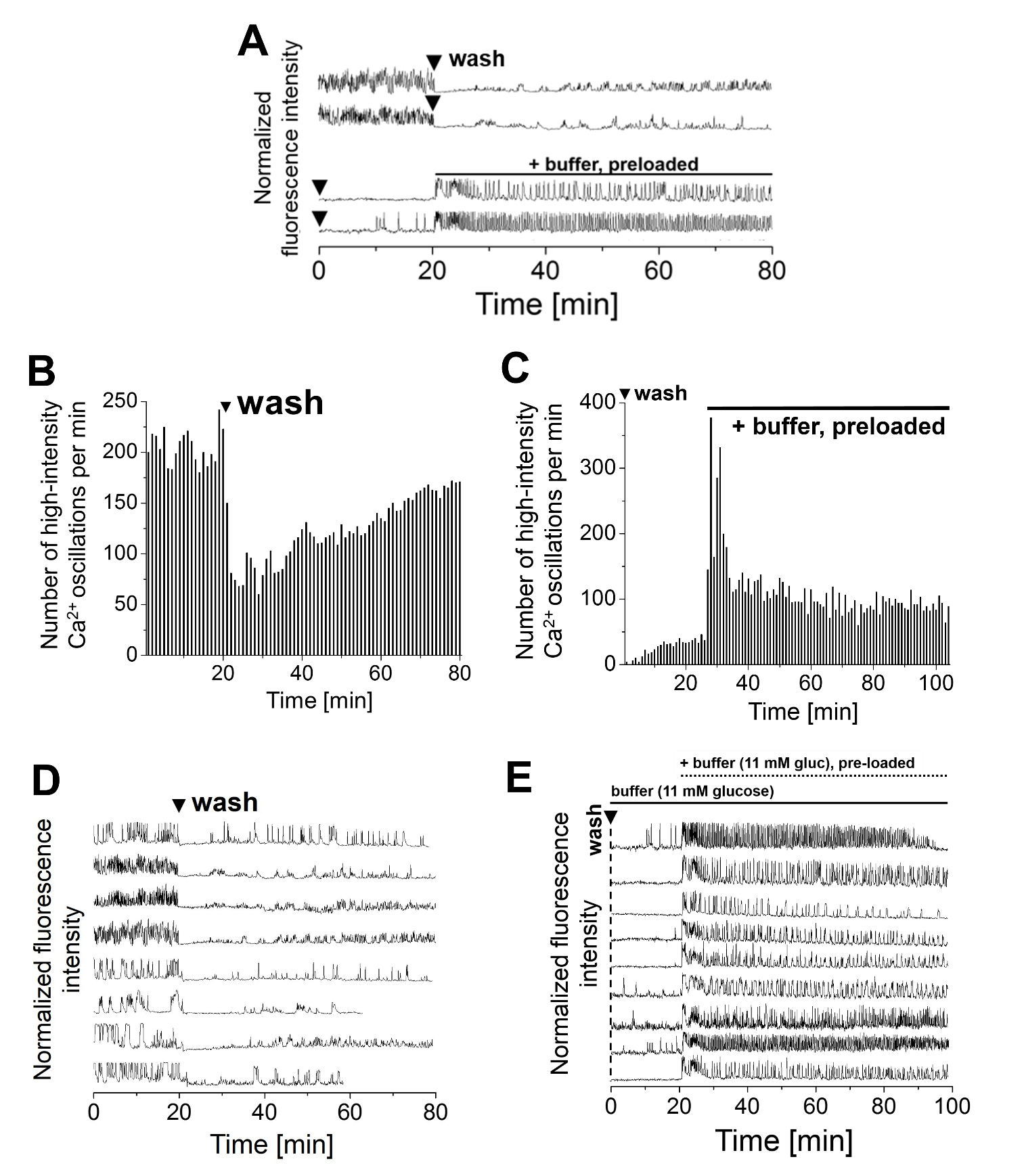
Figure S5. Endogenous (autocrine) signaling factors are essential for MIN6 and primary *β-*cell activity and insulin secretion. (A+B+D)** Stringent washing in a perfusion system (1.5 mL/min, indicated by ▼) reduced [Ca^2+^]_i_ oscillations in MIN6 cells, which gradually recovered during subsequent static incubation. **(A+C+E)** [Ca^2+^]_i_ oscillations of washed MIN6 cells immediately recovered upon addition of buffer that was pre-loaded on 2 x10^6^ MIN6 cells. Shown are representative single and averaged Ca^2+^ traces from MIN6 cells, stained with the Ca^2+^ indicator Fluo-4.


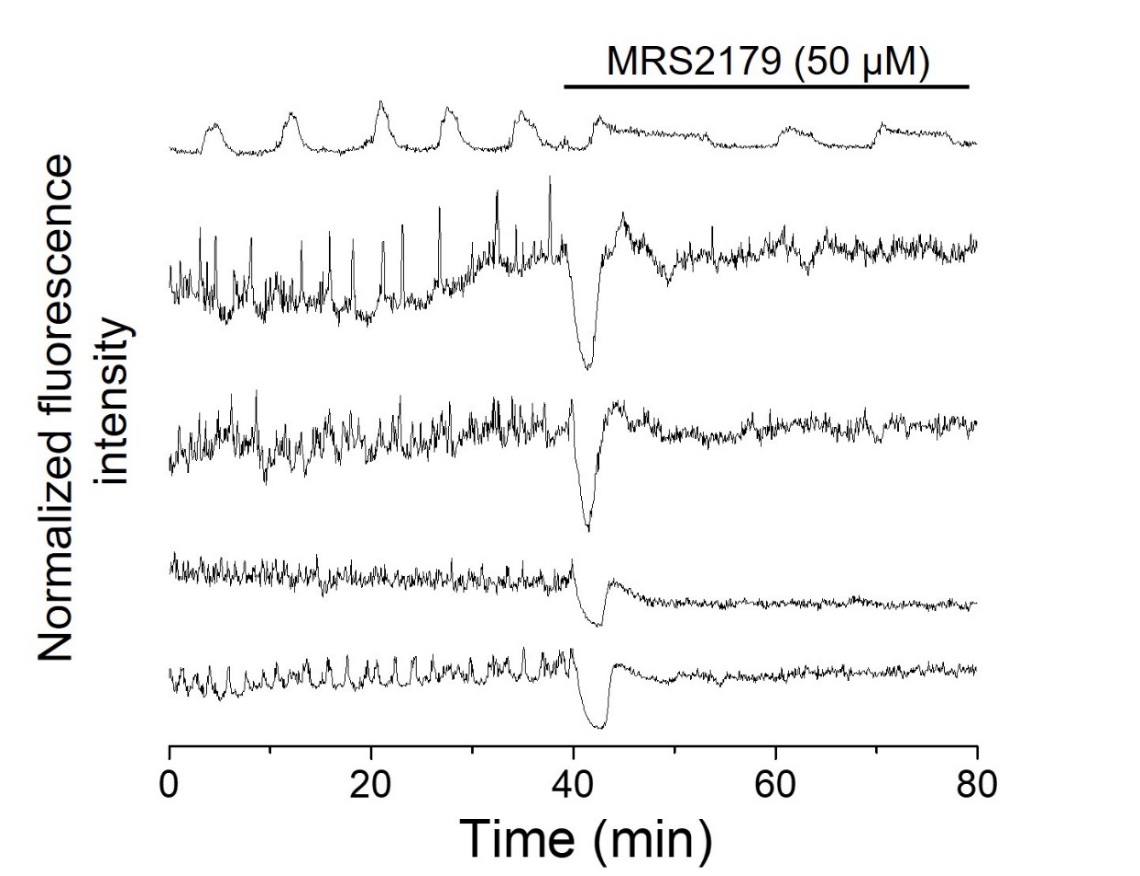


**Figure S6. Monitoring [Ca^2+^]_i_ within primary mouse *β*-cells in the presence of 11 mM glucose.** Mouse primary *β*-cells responded with high-frequency [Ca^2+^]_i_ oscillations to the presence of 11 mM glucose. Addition of MRS2179 (50 µM) reduced and partly stopped [Ca^2+^]_i_ oscillations. Imaging was performed in the presence of 11 mM instead of 5 mM glucose.


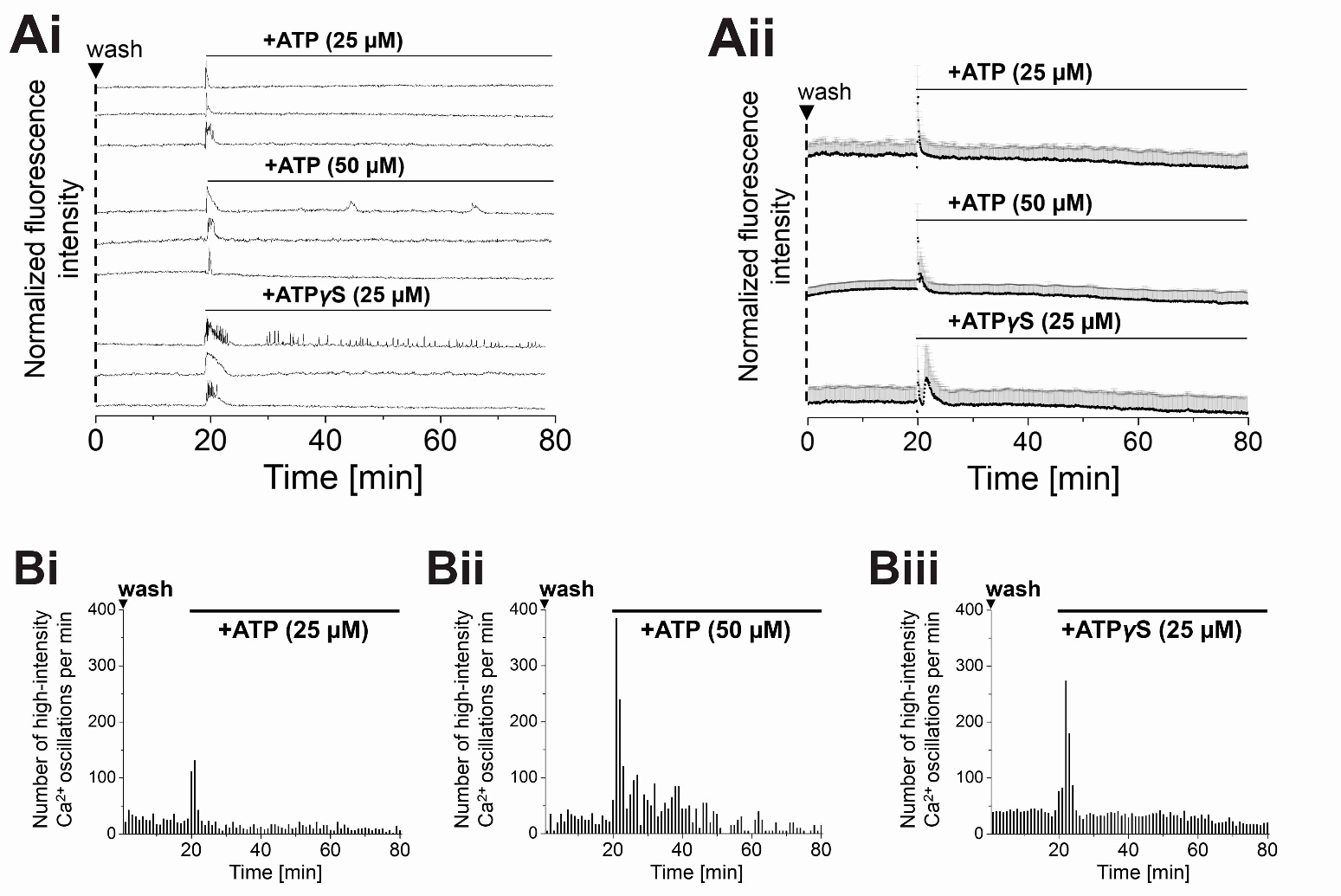


**Figure S7. Application of ATP to MIN6 cells in the presence of sub-stimulatory glucose evoked single [Ca^2+^]_i_ transients. (Ai)** Representative single and **(Aii)** average [Ca^2+^]_i_ traces from MIN6 cells, recorded with the Ca^2+^ indicator Fluo-4 in the presence of 3 mM glucose. **(Bi – iii)** Numbers of detected high-intensity [Ca^2+^]_i_ events per 60 s interval recorded from MIN6 cells. Shown are averages of n = 50 MIN6 cells. Addition of ATP and ATP*γ*S to pre-washed MIN6 cells induced single [Ca^2+^]_i_ transients.


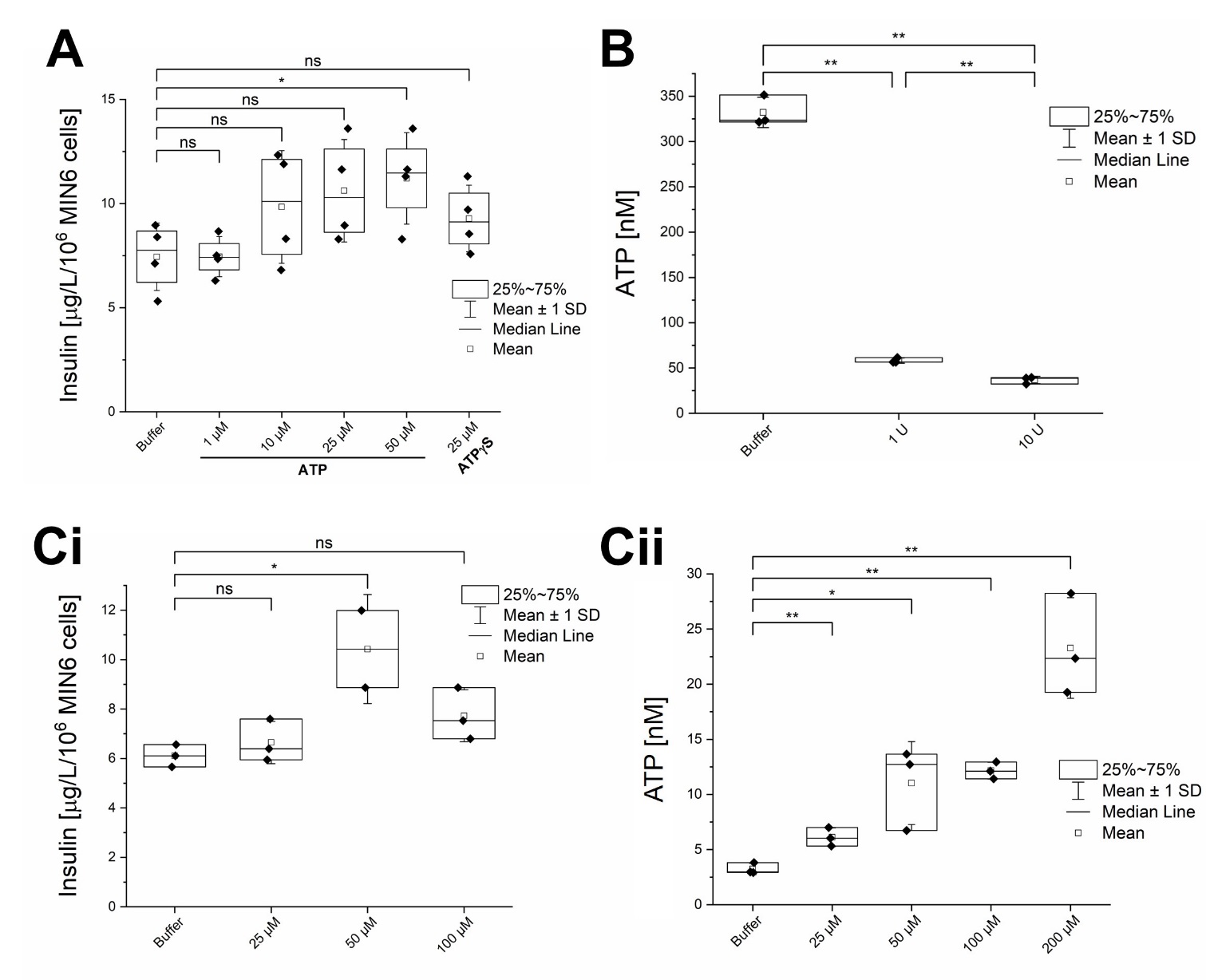


**Figure S8: Dot-plot representation of insulin secretion or extracellular ATP levels**. **(A)** Insulin secretion, as determined from MIN6 in the presence of ATP or ATPγS, corresponding to figure 1D. **(B)** Levels of endogenous ATP in the supernatant of MIN6 cells as determined in the presence of recombinant apyrase at different activities, corresponding to figure 2D. **(Ci)** Insulin and **(Cii)** ATP levels, as determined from the supernatant of MIN6 cells in the presence of ARL67156 in different concentrations, corresponding to figures 3Bi and Bii. Experiments were performed on MIN6 cells in the presence of 11 mM glucose.
